# Real-Time SPR Biosensing to Detect and Characterize Fast Dissociation Rate Binding Interactions Missed by Endpoint Detection and Implications for Off-Target Toxicity Screening

**DOI:** 10.3390/biom15060882

**Published:** 2025-06-17

**Authors:** William Martelly, Rebecca L. Cook, Chidozie Victor Agu, Lydia R. Gushgari, Salvador Moreno, Sailaja Kesiraju, Mukilan Mohan, Bharath Takulapalli

**Affiliations:** SPOC Proteomics, Inc., 19001 N. Scottsdale Road, Suite 285, Scottsdale, AZ 85255, USAchidoziea@spoc.bio (C.V.A.); lydiag@spoc.bio (L.R.G.); salvadorm@spoc.bio (S.M.); sailajak@spoc.bio (S.K.); mukilanm@spoc.bio (M.M.)

**Keywords:** sensor-integrated proteome on chip, off-target screening, kinetics, SPR biosensing, biomolecular interactions

## Abstract

Accurate detection of biomolecular interactions is essential in many areas, from the detection of the presence of biomarkers in the clinic to the development of therapeutic drugs and biologics in biopharma to the understanding of various biological processes in basic research. Traditional endpoint approaches can suffer from false-negative results for biomolecular interactions with fast kinetics. By contrast, real-time detection techniques like surface plasmon resonance (SPR) monitor interactions as they form and disassemble, reducing the risk of false-negative results. By leveraging cell-free expressed proteins captured on either glass or SPR biosensors and using two different commercial antibodies with variable off-rates that both target HaloTag antigens as a model, we compare and contrast results from a fluorescence endpoint assay versus real-time sensor-integrated proteome on chip (SPOC^®^) SPR-based detection. In this study, we illustrate the limitations of the representative immunofluorescent endpoint assay when investigating transient interactions characterized by fast dissociation rates. We highlight the importance of choosing reagents well suited to the selected assay, as well as the importance of considering binding kinetics and protein ligand conformational states when interpreting results from binding assays, especially for applications as critical as the off-target screening of therapeutics.

## 1. Introduction

Detection of biomolecular interactions is fundamental to applications in diagnostics, proteomics, and drug discovery. Diagnostics rely on the capture and detection of circulating biomarkers, proteins function within networks of interactions, and therapeutic drugs act by binding to specific targets. To screen for and detect these interactions, traditional methods have leveraged endpoint assays wherein a single measurement is taken after a series of incubations and reagent wash steps. However, molecular interactions are not static but are equilibrium reactions. They are instead driven by a dynamic balance between rates of association (*k*_a_) and dissociation (*k*_d_). Therefore, a critical limitation of endpoint assays revolves around the risk of false-negative results when attempting to detect interactions with fast binding kinetics. Such transient interactions may form, yet dissociate rapidly before detection. Endpoint assays are exposed to this limitation because they rely on the bound complex to be stable through a multitude of washing and secondary incubation steps in order for detection to be successful.

Interrogating transient interactions is of critical importance in drug discovery, where therapeutic specificity is paramount. While generally weaker than the intended on-target binding interactions, transient off-target binding interactions can be significant at elevated drug doses and elevated endogenous expression levels of off-targets in vivo [1]. Small molecule drugs for example have been estimated to interact with a minimum of ~6–11 unintended targets in the human body [2,3]. Even with therapeutic modalities that are considered less promiscuous like antibodies, investigations have identified that 33% of lead candidates exhibit off-target binding [4]. This lack of specificity has major implications for therapeutic efficacy and success rates. Approximately 75% of adverse drug reactions (ADRs) are due to dose-limiting toxicity which constrain therapeutic windows [5,6,7]. This toxicity occurs largely due to the interaction of drugs with off-target biomolecules, a problem contributing to an estimated 30% of drug failures.

In vitro promiscuity correlates with in vivo toxicity. Therefore, secondary pharmacological profiling assays for detecting interaction with a panel of targets most commonly associated with toxicity are required by regulatory guidelines for investigational new drugs and are critical in early-phase drug development [8,9,10]. Pharmaceutical companies employ a battery of secondary pharmacological profiling assays that often consist of panels of putative unsafe off-targets and rely on various radioligand- or fluorescent-based endpoint detection methods. Examples include the assays offered by the Eurofins Discovery portfolio, which are diverse and consist of panels of recombinant proteins, or the Charles River Retrogenix platform, consisting of arrays of cells over-expressing membrane proteins, to name a few [11,12,13].

While useful, these endpoint assays provide limited insight into the nature of any given interaction, offering only a narrow snapshot of bound complexes. Furthermore, these endpoint assays risk missing weaker off-target interactions that may nonetheless reduce safety and efficacy when applied in the clinic. Relying solely on such assays may be the difference between identifying a safe and effective therapeutic versus one with unforeseen off-target binding that fails Phase 1 trials.

To address these limitations, a variety of real-time and label-free biosensing approaches have been developed, including surface plasmon resonance (SPR) [14,15]. In contrast to endpoint assays, SPR can report the interaction with an analyte in solution without the need for fluorescent dyes or other reporter tags (label-free) and, importantly, as binding occurs (real-time), improving the detection of short-lived bound complexes. SPR has become a gold-standard technique for directly measuring the *k*_a_ and *k*_d_ of molecular interactions, which can be used to calculate occupancy times, bound complex half-life (*t*_1/2_), and the equilibrium dissociation constant (*K*_D_) [16,17].

By leveraging real-time assays in secondary pharmacological screening via techniques like SPR, the risk of false-negatives in off-target binding detection can be reduced, thereby improving the flagging of compounds or biologics with undesired off-target interactions in the early phases of drug discovery. SPR has long been leveraged in drug discovery pipelines to gain insights into drug–target binding interactions and now needs to be applied for the detection of undesired drug interactions.

Beyond off-target binding considerations, binding affinity characterization (*K*_D_) is also critical in drug discovery. When it comes to affinity, however, more is not always better. This has been the lesson learned in several burgeoning therapeutic modalities such as chimeric antigen receptor T-cell therapy (CAR-T), antibody drug conjugates (ADCs), and targeted protein degradation (TPD). Seven CAR-T cell therapies have been approved by the FDA since 2017, and it has been observed that moderate affinity of the antigen binding domain (*K*_D_ = ~50.0–100 nM range) correlates with antitumor efficacy of CAR-T therapies in the clinic [18,19]. While the first ADC to gain FDA approval was in the year 2000, next-generation designs have been created to overcome issues with dose-limiting toxicity, with nine out of the thirteen FDA-approved ADCs gaining approval recently in just the past five years [20]. In ADCs, reducing the affinity for target binding has been found to be a feasible strategy to improve efficacy, yielding increased tumoral diffusion and reduced on-target, off-site related toxicity [21,22]. TPD therapies orchestrate ternary complex formation between target proteins and native degradation effector molecules, like E3 ligases of the ubiquitin proteasome system. This ternary complex triggers choreographed processes that ultimately lead to the elimination of the target protein using natural processes [23]. As in CAR-T and ADC therapies, TPD-based therapies require precise affinity tuning to optimize efficacy. Higher affinity of TPD molecules can alter the binding dynamics towards non-functional binary interactions, shifting away from productive ternary complex formation and contributing to the well-recognized “hook effect” [24,25].

**Figure 1:**
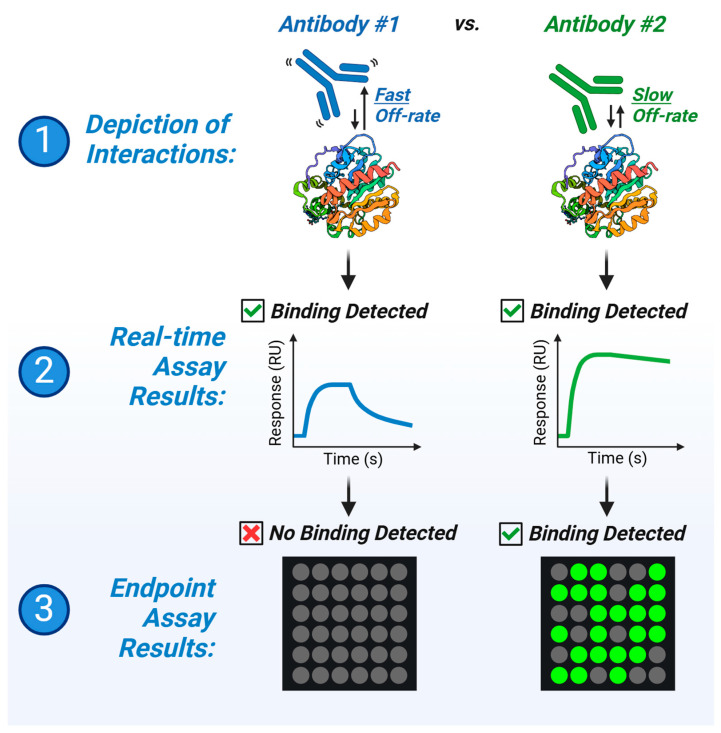
Graphical depiction of the observations from this study. Two anti-HaloTag antibodies were procured and screened for binding activity by two techniques: a fluorescent endpoint assay and a real-time surface plasmon resonance (SPR) assay. We observed that while binding of Antibody #2 (green) was detected in both the endpoint and real-time assays, binding of Antibody #1 (blue) was only readily detected in the real-time assay and not by initial endpoint screens. We demonstrate that compared with Antibody #2, Antibody #1 binding kinetics have a comparatively faster off-rate, limiting its detection in a traditional endpoint assay.

Sensor-integrated proteome on chip (SPOC^®^) is a next-generation protein biosensor technology enabling high density protein production directly onto SPR biosensors for cost-efficient and high-throughput real-time analyte screening [26]. SPOC leverages in vitro transcription and translation (IVTT) on proprietary Protein NanoFactory systems to synthesize various proteins of interest fused to a common HaloTag domain, used for in situ capture purification onto chloroalkane-coated SPR biosensor slides or glass slide substrates. By coupling cost-efficient cell-free protein synthesis for high-density on-chip protein libraries and label-free technologies like SPR, SPOC is poised to improve real-time biomarker screening, kinetic evaluation of therapeutic biologics and drugs, and basic research into protein interaction networks. In this study, we utilize two commercial antibodies raised against the common HaloTag protein tag to illustrate advantages of real-time screening. We find that fluorescent endpoint assay yields disparate binding results between these two antibodies when screening for successful capture of the IVTT protein spots on glass slide substrate. By contrast, real-time screening by SPR demonstrates that both antibodies are similarly capable of binding to the HaloTag fusion proteins present on the biosensor surface and contend that the different kinetic profiles exhibited by these two antibodies result in biased, false-negative binding results when screening using a traditional fluorescent endpoint assay (Figure 1). Furthermore, we highlight how SPOC technology enhances the multiplex capacity of SPR screening, yielding up to ~864 protein ligand spots in our custom LSA^XT^ Carterra instrument (a ~2.2-fold increase from the standard 384 commercial instrument capacity).

## 2. Materials and Methods

### 2.1. Antibodies

Anti-HaloTag (Antibody #1) was sourced in phosphate-buffered saline (PBS) only format from Proteintech (Rosemont, IL, USA; 28a8) while the polyclonal rabbit anti-HaloTag (Antibody #2) was sourced from Promega (Madison, WI, USA; G9281). Goat anti-mouse (115-165-062) and anti-rabbit (111-165-003) Cy3-labeled secondary antibodies were sourced from Jackson ImmunoResearch (West Grove, PA, USA). In fluorescent assays, all primary antibodies were diluted into 1X PBS containing 0.2% Tween-20 and 5.0% fat-free milk (PBST-M).

### 2.2. In Vitro Transcription and Translation of SPOC Arrays

Capture of the IVTT SPOC arrays was performed as described previously [27]. Briefly, plasmid DNA containing HaloTag fusion protein open-reading frames and compatible with cell-free expression were sourced from DNASU plasmid repository and printed into nanowells of a nanowell slide. The nanowell slide was then affixed to SPOC Proteomics’ in house built and proprietary Protein NanoFactory systems (previously AutoCap) along with either glass or biosensor capture slide substrates, depending on the assay. HeLa IVTT cell-free extract (ThermoFisher, Waltham, MA, USA; 8882) was prepared and injected over the nanowell slide surface, followed by press sealing of the nanowells against the respective capture slide surfaces. This sandwich was incubated at 30 °C for at least 2.0 h prior to disassembly and rinsing of nanowell and capture slides in 1X PBS supplemented with 0.2% Tween-20 (PBST). Capture slide surfaces were subsequently used for follow-on fluorescent or SPR assays.

### 2.3. Fluorescent Assay

Hydrogel-coated partially activated glass capture slides were purchased from Schott (Mainz, Germany; 1070936). Amine-terminated HaloTag ligand was purchased from Iris Biotech GmbH (Marktredwitz, Germany; RL-3680). Amine-terminated HaloTag ligand was prepared at 1.0 mg/mL concentration and 80 µL of the solution was pipetted onto a clean lifter slip followed by placement of the glass capture slide facing down onto the solution to react the activated hydrogel surface with the HaloTag ligand. The glass capture slide was incubated on ligand overnight at room temperature and subsequently quenched/blocked in SuperBlock from ThermoFisher (37516) for at least 30 min at room temperature with rocking. Blocked capture slides were then used for capture of IVTT proteins as described above.

All antibodies were diluted into PBST-M. Primary antibodies were diluted 1:750 and the glass slides harboring captured HaloTag fusion proteins were exposed to primary antibody for 1.0 h at room temperature with rocking. After rinsing slides in PBST several times, secondary antibody was diluted at a 1:500 dilution and incubated over the capture slide for an additional 1.0 h at room temperature. The capture slide was then rinsed in PBST followed by diH_2_0 and subsequently dried with compressed air before fluorescent scanning in an InnoScan 910 AL Microarray Scanner (Innopsys, Carbonne, France). The slide was first probed with Antibody #1, followed by initial scanning and subsequent re-probing with Antibody #2.

### 2.4. Surface Plasmon Resonance Assay

SPR running buffer (1X PBS, 0.2% BSA, 0.05% Tween-20, pH = 7.4) was prepared fresh, filtered using a 0.2 µm bottle top filter (Thermo Fisher Scientific, Waltham, MA, USA; 595-4520), and degassed in a vacuum chamber for at least 20 min. The custom LSA^XT^ instrument (Carterra, Salt Lake City, UT, USA) was primed in this running buffer prior to biosensor docking. The SPOC biosensor harboring IVTT HaloTag fusion proteins was rinsed in 1X PBS and the back, non-gold coated side of the sensor was dried on a lint-free cloth while keeping the top wet. The sensor was quickly mounted onto an LSA^XT^-compatible, bare prism cartridge using 12.0 μL of refractive index-matching mounting oil (Cargille, Cedar Grove, NJ, USA; 19586) and subsequently introduced into the instrument. Running buffer sufficient to coat the top of the slide was pipetted on top of the mounted biosensor to ensure proteins were kept wetted and to enable efficient docking of the instrument’s single flow cell. A priming injection of running buffer was performed to purge any trapped bubbles that may have formed during flow cell docking. Ligand-containing ROIs and reference ROIs were selected followed by analyte screening. The flow cell was set to 21 °C and Antibody #1 and #2 were diluted 1:50 in running buffer (133.3 nM), and the screen was performed with 15 min association and 30 min dissociation times. Injection of 10.0 mM Glycine-HCl (pH = 2.4) regeneration buffer injections were performed after initial screens with the antibodies to assess the impact of acid wash on antibody binding.

### 2.5. Data Analysis

**Figure 2:**
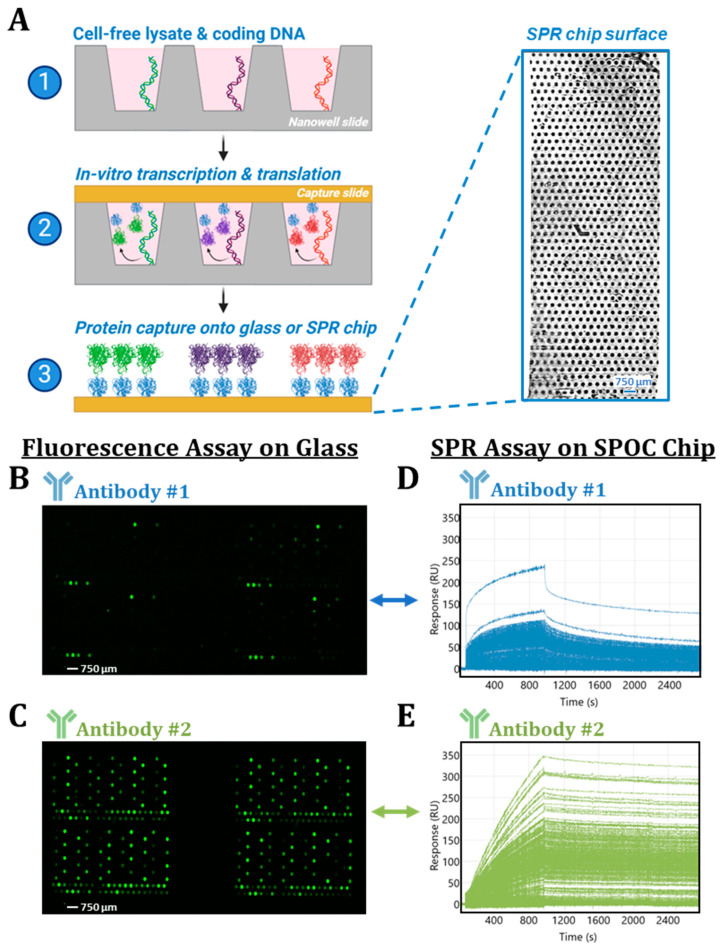
Results from fluorescent-based endpoint detection and SPR-based real-time detection of IVTT-expressed HaloTag fusion proteins captured onto glass or SPOC chip capture slide substrates. (**A**) Graphical depiction of the workflow for producing SPOC biosensors via the Protein NanoFactory system. To the right, an image of the SPOC biosensor surface from within the custom Carterra LSA^XT^ SPR instrument flow cell is shown. The array of spots contain various HaloTag fusion proteins covalently captured to the biosensor surface and the hockey stick-shaped fiducial mark enables orientation of the array and identification of HaloTag protein-containing spots. To assess the extent of HaloTag fusion protein capture on glass, an arrayed glass slide was probed with Antibody #1 and detected with Cy3-labeled goat anti-mouse secondary antibody (**B**). The same slide was again probed with Antibody #2, followed by detection using Cy3-labeled goat anti-rabbit secondary antibody (**C**). After each secondary antibody incubation, the slide was scanned on an InnoScan 9100 microarray imager. Presence of a green fluorescent signal at discrete spots indicates detection of HaloTag fusion proteins by the respective primary antibodies. Only two of the four total subarrays present on the glass slide (see Figure A1 for the full glass slide scans) are shown. Sensorgrams of the binding signal obtained from 355 spots across the biosensor surface during (**D**) Antibody #1 (blue) and (**E**) Antibody #2 (green) injections. Both antibodies were injected at 133.3 nM concentration over the course of a 15 min association phase (t = 0.0–900 s), followed by a 30 min dissociation phase (t = 901–2700 s). Each kinetic trace represents the binding signal observed from a single region of interest (ROI) or ligand selected for analysis on the SPOC array. Individual sensorgrams from eachROI reported here are displayed without overlay in Figure A2.

Raw data from the Carterra LSA^XT^ were analyzed in Kinetics analysis software (Carterra, v1.9.0.4167). Data were y-aligned and double-referenced using blank running buffer-only injections and reference, control ROIs where HaloTag fusion protein was not expected to be captured. Once pre-processed, all data were fitted with a 1:1 Langmuir binding model to obtain off-rate kinetic parameters. Because the analytes used in the assay are bivalent, the data are complicated by avidity effects, and therefore, the 1:1 binding model is acknowledged to yield only an approximation of the affinity for each antibody. The polyclonal nature of the rabbit anti-HaloTag antibody also complicates the analysis. Multiple clones are likely contributing to the SPR binding signal observed for the rabbit antibody, and the reported off-rates describe the aggregate rate observed from this polyclonal analyte. Prism 9.2.0 (332) was utilized to generate reported statistical parameters, dot plots, and scatter plots. Variation in the observed anti-HaloTag antibody binding magnitudes (R_max_) reflect the inherent capture level variation obtained by our protein capture strategy (Figure 2A). The capture level in our system can be impacted due to the variations in sub nano-liter volume of DNA printed in nanowells and the different sizes, transcription and translation efficiencies, and biochemistry of each unique HaloTag fusion protein, which can impact overall yield from the IVTT-based nanowell expression. The R_max_ values obtained for the various spots across the sensor are directly plotted in the scatter plots throughout the manuscript and provide a direct sense of the observed variability in our assay.

## 3. Results

The proteins captured on the biosensors or glass used in this study were expressed from plasmid DNA printed in nanowell slides by IVTT via the Protein NanoFactory system (previously referred to as AutoCap instrument) (Figure 2A) [27]. All proteins were expressed fused to a common tag (HaloTag) that is used to covalently capture IVTT fusion proteins onto HaloTag ligand (chloroalkane)-coated capture slide substrates [28]. The choice of capture slide substrates is modular on the Protein NanoFactory system and in this study includes capture of proteins onto glass slides (in the case of fluorescent endpoint screening) or SPR biosensors (in the case of real-time screening). For quality control purposes, we have been utilizing two different commercially sourced antibodies to confirm capture of HaloTag proteins on glass and SPR slides. Both antibodies perform consistently in their respective assays and enable us to assess and validate protein expression and capture. Reagents that are high quality, well-characterized, and utilized to characterize important assay metrics like this are known as “tool reagents” or “tool antibodies.” During internal testing and validation, we observed disparate results with these two commercial reagents on glass and SPR slides, which provided an opportunity to illustrate the advantages of real-time screening over traditional, fluorescent-based endpoint strategies when interrogating transient interactions.

### 3.1. Disparities in Fluorescent-Based Endpoint Detection

**Figure A1:**
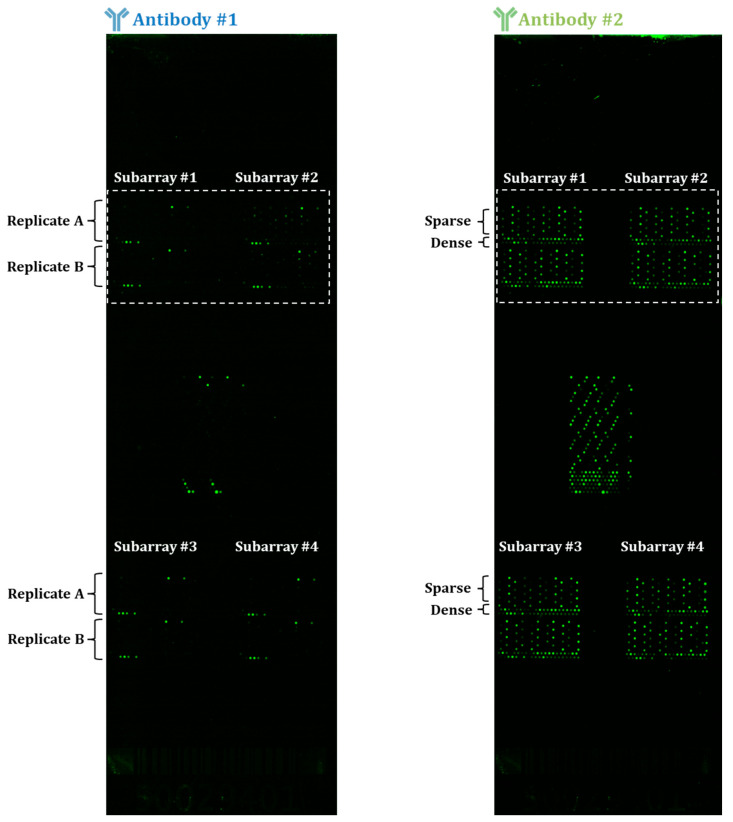
Full scan of the IVTT HaloTag fusion protein spotted glass slide probed with anti-HaloTag Antibody #1 (slide scan on the left) and Antibody #2 (slide scan on the right). Subarrays #1 and #2 are displayed in Figure 2 (area contained by the dashed white box). Each of the four subarrays included in this analysis contain two replicate sets (A and B) printed to enable capture of IVTT expressed HaloTag protein ligands within a sparsely and densely spotted region. The same glass slide was re-probed directly with Antibody #2 primary after initially probing with Antibody #1, without exposing the capture surface to any stripping or regeneration buffer solution. For the purposes of this manuscript, the array in the center of the slides was excluded from consideration, though the same disparity between the antibody binding patterns emerges.

The antibodies compared in this analysis include a monoclonal, mouse-derived anti-HaloTag antibody obtained from Proteintech (28a8) that we will refer to as “Antibody #1”, which was tested alongside a polyclonal, rabbit-derived anti-HaloTag antibody obtained from Promega (G9281) that we will refer to as “Antibody #2”. IVTT HaloTag fusion proteins were expressed across four identical subarrays captured at distinct areas across a HaloTag ligand-coated glass capture slide. Within each subarray, a set of proteins were expressed in two replicate sets (A and B), and within each replicate set, proteins were captured in a “sparse” or a “dense” region (Figure A1). Following HaloTag protein expression, the glass slide was probed with Antibody #1 followed by Antibody #2 anti-HaloTag antibodies to determine if protein capture was successful.

The glass capture slide was first incubated with Antibody #1 followed by incubation with a Cy3-labeled goat anti-mouse secondary antibody and subsequent fluorescent scanning to determine the extent of the bound primary antibody. Using a fluorescent endpoint assay, Antibody #1 appears to only bind a fraction of HaloTag fusion proteins (6 out of 86 per subarray; ~7%) anticipated to be captured in the replicate arrays (Figure 2B). At first glance, the results suggest that protein capture may have been unsuccessful, or that Antibody #1 is only capable of binding to and recognizing a small fraction of the HaloTag fusion proteins present.

Following the initial slide scanning after Antibody #1 incubation and detection, the glass capture slide was re-introduced into wash buffer and was similarly probed with Antibody #2. After Antibody #2 incubation, a Cy3-labeled goat anti-rabbit secondary antibody was added and subsequently washed and scanned as before. In contrast to Antibody #1, Antibody #2 confirmed the presence of most of the expected HaloTag fusion proteins (84 out of 86 per subarray; ~98%) captured across each subarray, revealing the expected sparse and dense pattern of protein spotting by a fluorescent endpoint assay (Figure 2C). Results from Antibody #2 probing confirmed that the expression and capture of the HaloTag fusion proteins on the glass slide substrate was indeed successful. Therefore, in the case of the Antibody #1 assay, lack of protein expression or capture cannot explain the detected signal observed from initial screening with Antibody #1. Alternatively, Antibody #1 may have faster binding kinetics than Antibody #2, preventing efficient detection by the secondary antibody for the majority of HaloTag protein ligands. The results from endpoint fluorescent assay suggest that Antibody #2 would serve as a better tool reagent for validating and characterizing protein expression and capture efficiencies of the IVTT protein arrays. However, it cannot be ruled out that the secondary antibody for Antibody #1 is also not the culprit at this point.

### 3.2. Insights from Label-Free Kinetic Screening

For label-free screening via SPOC, an analogous IVTT HaloTag fusion protein array was captured onto a HaloTag ligand-coated SPR biosensor surface followed by mounting of the biosensor onto a custom Carterra LSA^XT^ prism cartridge using a refractive index mounting oil. An array pattern revealing distinct spots across the biosensor surface is visible after docking of the flow cell and priming of the running buffer over the sensor (Figure 2A). This spot pattern is indicative of successful SPOC array HaloTag fusion protein capture. SPOC is capable of forming an array of ~864 spots (18 × 48) in the custom LSA^XT^ instrument analysis area, enhancing the multiplexity of the base technology ~2.2-fold as the instrument allows typical commercial users to screen up to 384 interactions. We currently operate a custom LSA^XT^ instrument using Carterra’s Navigator software (v2.1.2.3416) that is limited to addressing up to 384 spots on the SPOC array. We are currently working on enabling the full simultaneous readout and analysis of 1000 protein spots to make use of the full capacity of the SPOC biosensor. Ultimately, we hope to vastly expand the number of interactions that can be analyzed by SPR today. Utilizing the custom LSA^XT^, we have already demonstrated the ability to produce and screen a chip with up to 2400 ligands [27]. A hockey stick-shaped mark in the center of the biosensor serves as a fiducial to orient users for correct identification of spots containing HaloTag fusion protein ligands (Figure 2A). Using a customized version of Carterra’s Navigator software, these spots can then be selected as regions of interest (ROIs) for tracking of the SPR binding signal during analyte screening.

After ROI labeling, the SPOC biosensor was first probed with Antibody #1 followed by several blank running buffer-only injections and subsequent injection of Antibody #2. In SPR and other real-time assays, interactions are monitored as they occur. The output from the SPR screen is double-referenced and the signal (response units; RUs) measured at each ligand is plotted over time (seconds), generating a sensorgram [29]. In this experiment, the antibodies were each diluted to 133.3 nM in running buffer and injected over the SPOC biosensor for a 15 min association phase (t = 0.0–900 s), followed by 30 min of running buffer-only flow in the dissociation phase (t = 901–2700 s). Increases in RU over the course of the association phase in the double-referenced sensorgrams reflect binding events of analyte to the ligands on the sensor surface. Each kinetic trace within the sensorgram represents the binding signal observed from a single ROI or ligand, and the signals from each of the 355 ROIs analyzed in this dataset are shown as an overlay.

**Figure A2:**
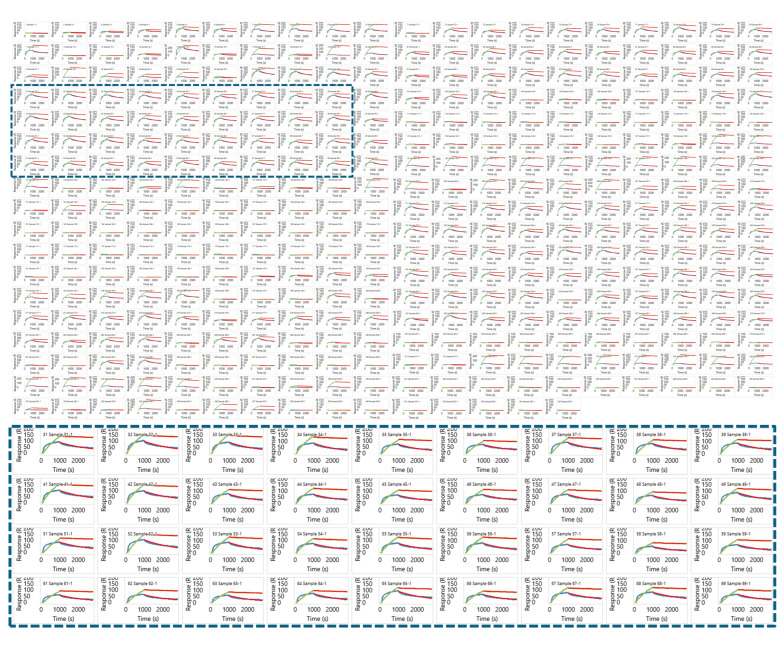
Individual sensorgrams of binding signal at each HaloTag protein ligand from the entire 355 ROI array that are displayed as an overlay in Figure 2D,E. The kinetic data at each ROI are shown, with Antibody #1 binding signal being colored blue and Antibody #2 binding signal being colored green. The 1:1 binding kinetic model used for obtaining off-rate (*k*_d_) parameters and generating the dot plot (Figure 3) is overlaid on the traces and indicated by the red line. A representative sub-set of the data are blown up larger beneath the 355 sensorgrams and is indicated by the blue dashed outline.

The sensorgrams generated over the course of Antibody #1 (Figure 2D) and Antibody #2 (Figure 2E) anti-HaloTag injections are reported. Both anti-HaloTag antibodies are observed to bind the HaloTag fusion proteins on the biosensor surface but with quantifiable and visually discernable differences in their binding magnitudes (R_max_) and kinetics, as shown in their respective sensorgrams. Antibody #1 (blue kinetic traces) produced lower R_max_ binding responses (up to ~150–250 RU) and had fast association and dissociation kinetics, reaching an approximate steady-state equilibrium within ~10 min of sample injection and dissociating rapidly from the ligands once buffer flow is restored in the dissociation phase (Figure 2D). By contrast, Antibody #2 (green kinetic traces) produced much higher R_max_ binding responses (up to ~350 RU) and had comparably slower binding kinetics. Unlike Antibody #1, Antibody #2 does not reach a discernable steady-state equilibrium over the course of the 15 min association phase, and there is negligible dissociation of the antibody once bound to the ligands after running buffer flow is restored during dissociation (Figure 2E). The results from the SPR screening reveal that Antibody #1 is capable of binding to more of the HaloTag protein ligands than observed in the fluorescent endpoint assay. Binding responses from both antibody injections against all individual ROIs are shown overlayed in Figure 2, and sensorgrams for each ROI individually are reported in Figure A2.

### 3.3. Fast Kinetics of Antibody #1 Partially Underlie Disparities in Endpoint Assay Results

**Figure 3:**
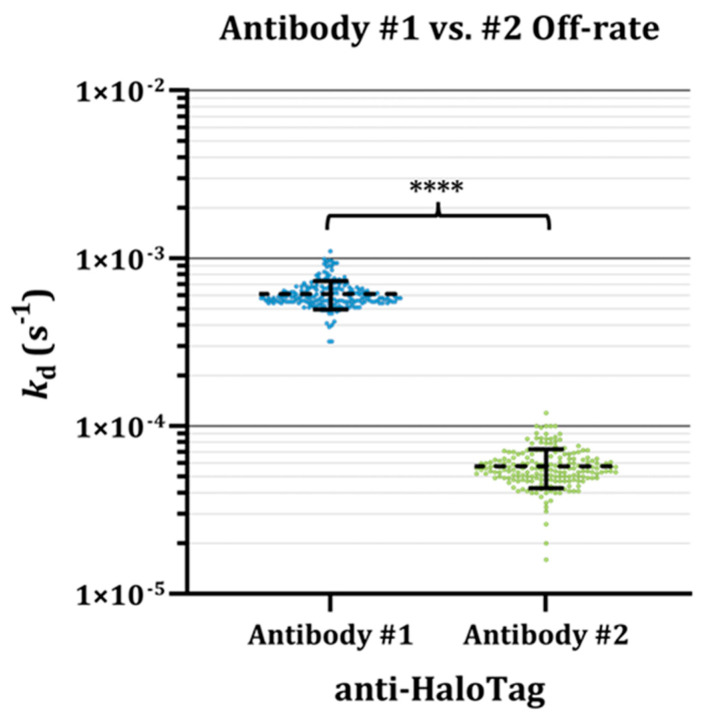
Antibodies #1 and #2 have distinct off-rates. Dot plot of off-rate kinetic values (*k*_d_) obtained after Antibody #1 (blue) or Antibody #2 (green) were injected over the sensor and allowed to dissociate for 30 min. Each dot represents the *k*_d_ measured at an individual HaloTag ligand containing a spot on the SPOC biosensor, with the mean (dashed line) and standard deviation (error bars) overlayed (n = 197, **** = *p* < 0.0001).

Leveraging the advantages of real-time screening in resolving the binding kinetics of interactions, the dissociation rate of Antibodies #1 and #2 against the HaloTag protein ligands was estimated by applying a 1:1 binding model to the dissociation phase of the analyte injections (Figure A2). The analysis reveals that Antibody #1 has an order of magnitude faster average dissociation rate (*k*_d_ = 6.13 × 10^−4^) compared with Antibody #2 (*k*_d_ = 5.77 × 10^−5^) (Figure 3). An informative metric that can be gleaned from the dissociation rate is the bound complex half-life (*t*_1/2_), which can be obtained by taking the natural log of 2 over the dissociation rate ($t_{1/2}=\frac{\ln\left(2\right)}{k_{d}}$) [30]. Using the average *k*_d_ obtained for both antibodies (Figure 3), we estimate that while Antibody #1 has a *t*_1/2_ = ~18.8 min, Antibody #2 has a 10.2-fold longer, more stable complex with *t*_1/2_ = ~200.2 min. These insights from the label-free kinetic assay support the hypothesis that the faster binding kinetics of Antibody #1 underlie the lack of HaloTag protein binding detection in the fluorescent endpoint assay (Figure 2B).

Due to the polyclonal nature of Antibody #2, this analyte is a mixture of multiple antibody clones that each contribute independently to the binding signal. Therefore, it must be acknowledged that the observed binding signal and subsequent off-rate kinetic estimates for Antibody #2 are due to the binding of a combination of antibody clones. Furthermore, because the concentration of any individual antibody in the polyclonal sample is unknown, the on-rate cannot reasonably be modeled. The off-rate by contrast is a concentration-independent measure and is often modeled in scenarios where the analyte concentration in a sample is unknown, as in off-rate screening regimens [31,32,33]. We have observed that kinetic rates can be estimated consistently, and are independent of, protein ligand capture levels [26,34]. Therefore, while capture levels vary from spot to spot as seen by the observed variable anti-HaloTag binding level magnitudes at different ROIs (Figure 2), consistent kinetic binding rates can be extracted. Finally, because both Antibody #1 and Antibody #2 are bivalent analytes, the interaction kinetics are better characterized by avidity instead of affinity. The binding responses contain inherent kinetic heterogeneity that cannot be accurately accounted for by the 1:1 binding model applied, and hence, the off-rates reported here are acknowledged to be estimates.

### 3.4. Acidic Buffer Treatment Drastically Enhances Antibody #1 Detection

While the results from the endpoint screen (Figure 2B) seemed to suggest that Antibody #1 was only capable of binding a small fraction of the immobilized HaloTag fusion protein ligands, the results from the real-time screen (Figure 2D) revealed that there is a significant amount of Antibody #1 binding detectable to a large portion of the ligands present on the SPOC biosensor. Results from the real-time assay further revealed a significantly high off-rate inherent to Antibody #1 (Figure 3), likely underlying the lack of binding detected in the endpoint assay compared with Antibody #2. Interestingly, observations from regeneration buffer screening revealed another factor influencing the detection efficiency of Antibody #1 by an endpoint assay [27].

Interestingly, when performing regeneration buffer screening assays, we observed that Antibody #1 binding signals would initially drastically increase in magnitude upon the first instance of biosensor regeneration with 10 mM Glycine-HCl (pH = 2.4). Binding signals would then remain consistently elevated upon subsequent re-injections of Antibody #1, prompting us to investigate further.

**Figure 4:**
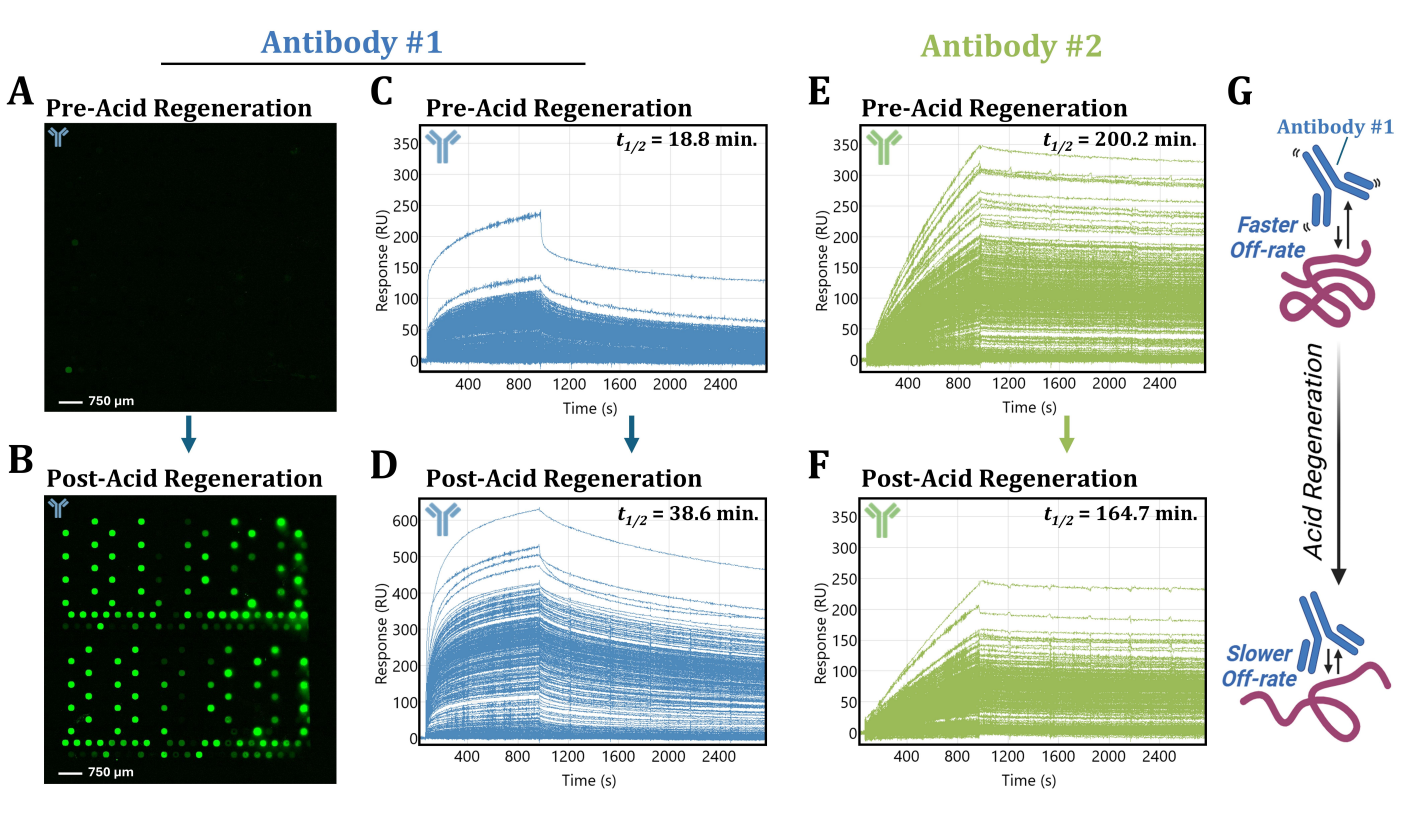
Effect of acidic buffer regeneration treatment on the detection of Antibody #1 and #2 binding. A glass microscope slide with arrayed HaloTag protein was prepared and probed with Antibody #1 and subsequent Cy3-labeled goat anti-mouse secondary antibody for detection before (**A**) or after (**B**) the arrays were exposed to a 5.0 min acid wash with 10 mM Glycine-HCl (pH = 2.4). Sensorgram of binding signal obtained from 355 spots across a HaloTag ligand spotted biosensor surface over the course of Antibody #1 injection before (**C**) and after (**D**) the biosensor had been regenerated with the 10 mM Glycine-HCl (pH = 2.4) acidic regeneration buffer. Sensorgram of binding signal obtained from 355 spots across the biosensor surface over the course Antibody #2 injection before (**E**) and after (**F**) the biosensor had been regenerated. Note that both panels (**C**,**E**) are also reported in Figure 2D and Figure 2E, respectively, but are reproduced here for ease of comparison to post-acid regeneration data. Each kinetic trace in all sensorgrams represents the binding signal observed from a single ROI selected for analysis on the SPOC array. A graphic representation (**G**) of the proposed role that acid regeneration may have in revealing a cryptic epitope in the HaloTag protein, leading to subsequent enhancement of Antibody #1 binding is shown.

**Figure A3:**
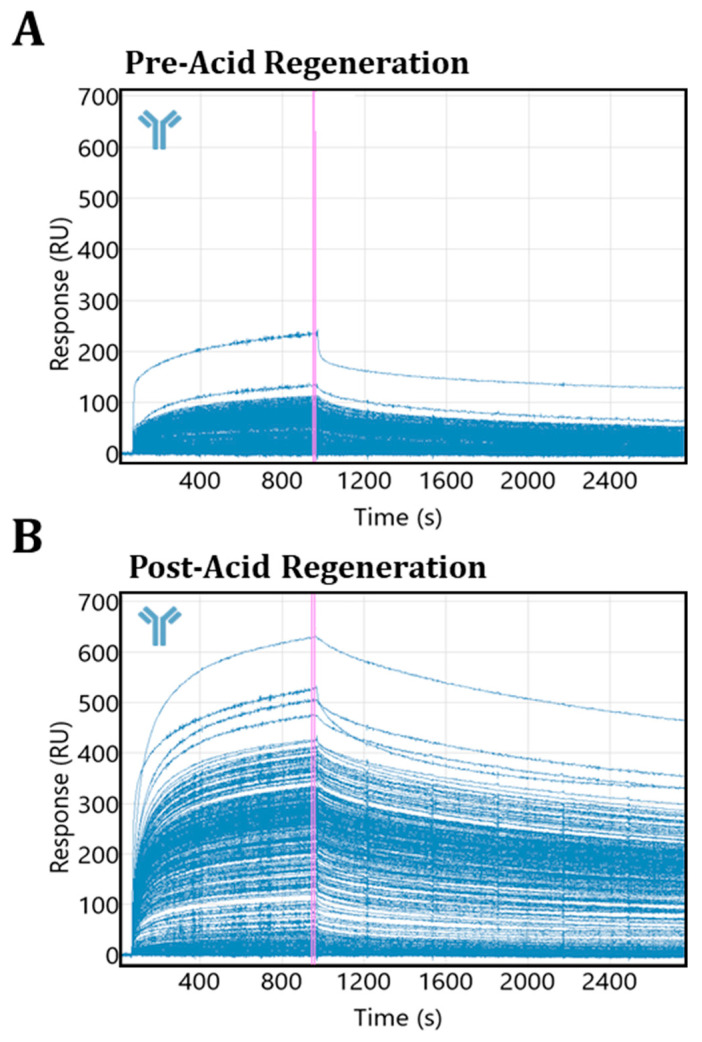
Thresholds used for obtaining R_max_ values for ligand binding comparative analysis. Sensorgrams of the binding signal obtained from 355 spots across the biosensor surface during Antibody #1 injection pre- (**A**) or post- (**B**) acid regeneration with 10 mM Glycine-HCl (pH = 2.4). Average signal measured within the bounds of the thresholds (pink vertical lines) were collected for each ligand, during both antibody injections, and utilized to generate the scatter plot in Figure A4A. Similar thresholds were set for Antibody #2 and used for the scatter plot shown in Figure A4B.

**Figure A4:**
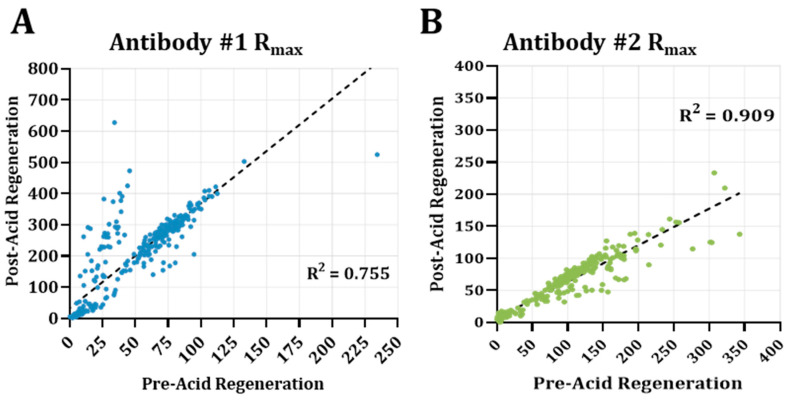
Effect of acidic regeneration on anti-HaloTag binding signal. Scatter plot comparing R_max_ values measured from 355 spots across the HaloTag protein spotted biosensor surface for both (**A**) Antibody #1 and (**B**) Antibody #2 injections are reported, comparing values measured before and after sensor regeneration with the acidic 10 mM Glycine-HCl (pH = 2.4) regeneration buffer.

In a fluorescent endpoint assay, when the captured HaloTag protein array was probed immediately, prior to exposure to the acidic 10 mM Glycine-HCl (pH = 2.4) buffer, we observed negligible Antibody #1 binding as before (Figure 4A). However, after a brief 5.0 min incubation in the acidic regeneration buffer, binding of Antibody #1 to the vast majority of the ligands present on the array (82 out of 86 per subarray; ~95%) can be successfully detected by fluorescent endpoint assay (Figure 4B). Through a label-free assay, the effect of acidic buffer regeneration was similarly drastic (compare Figure 4D with Figure 4C), yielding a large increase in R_max_ binding response measured for Antibody #1 (up to ~600 RU) over the course of the 15 min injection. The R_max_ values observed for each ROI at the end of association phase pre- and post-acid regeneration were obtained (Figure A3) and plotted against each other in a scatter plot to assess the similarities in HaloTag protein detection under each condition (Figure A4A). While there were numerous HaloTag protein ligands that were more readily detected in the label-free assay only after 10 mM Glycine-HCl (pH = 2.4) regeneration, there was large overlap in detectable hits between the two conditions in the real-time screen, producing a moderate correlation of R^2^ = 0.755 (Figure A4A). This enhancement effect was not observed for Antibody #2. In contrast to Antibody #1, 10 mM Glycine-HCl (pH = 2.4) acid regeneration led to a slight reduction in the magnitude of the overall Antibody #2 binding response (compare Figure 4F with Figure 4E) without impacting overall HaloTag protein ligand detection as the Antibody #2 R_max_ hits pre- and post-regeneration were very similar producing an R^2^ = 0.909 (Figure A4B).

**Figure A5:**
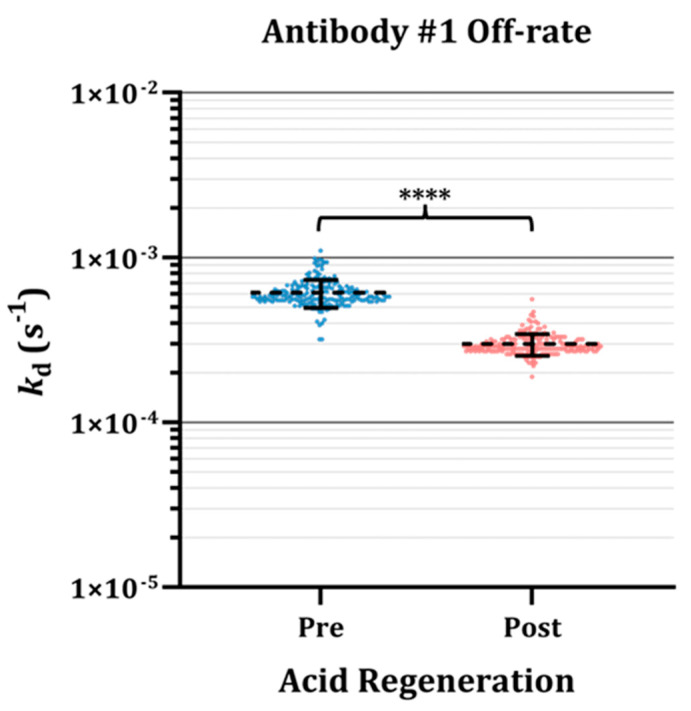
Antibody #1 off-rate pre- and post-acid buffer regeneration. Dot plot of off-rate kinetic values (*k*_d_) obtained after Antibody #1 was injected over the sensor and allowed to dissociate for 30 min before (blue) and after (red) acid regeneration with 10 mM Glycine-HCl (pH = 2.4). Each dot represents the *k*_d_ measured at an individual HaloTag ligand on the SPOC biosensor, with the mean (dashed line) and standard deviation (error bars) overlayed (n = 197, **** = *p* < 0.0001).

The exposure of the HaloTag protein ligands to the acidic buffer is likely inducing a conformational change in the HaloTag protein that reveals a cryptic epitope, enabling more efficient binding of Antibody #1 (Figure 4G). Additionally, the *k*_d_ for Antibody #1 after acid regeneration was significantly reduced (2.99 × 10^−4^) (Figure A5), yielding a 2-fold more stable half-life of *t*_1/2_ = ~38.6 min compared with the half-life estimate for Antibody #1 pre-regeneration. By contrast, the half-life of Antibody #2 reduced slightly by 0.82-fold post-regeneration to *t*_1/2_ = ~164.7 min. These results indicate that a combination of 1) the fast off-rate and 2) the conformational state of the HaloTag protein contributes to the disparities initially observed in the endpoint assay between Antibodies #1 and #2.

**Figure 5:**
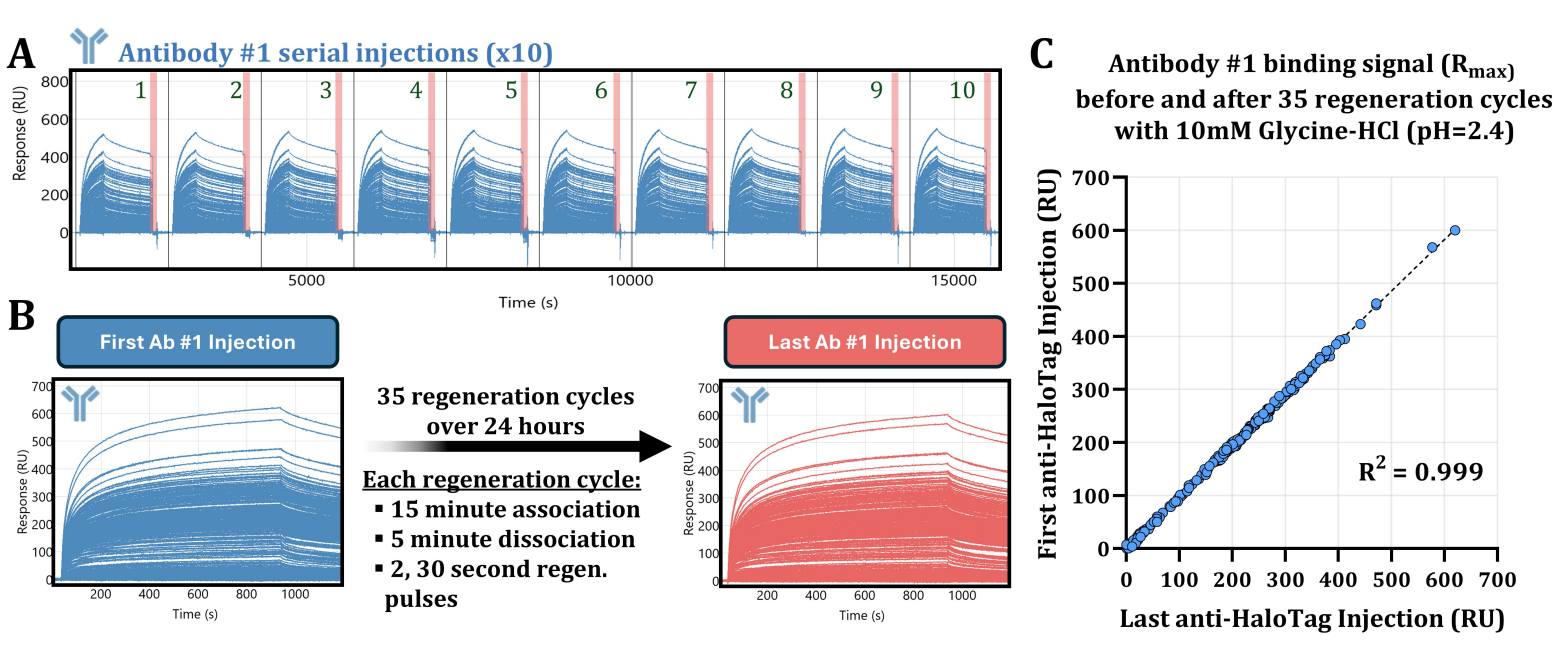
Covalent capture of HaloTag ligands enables SPOC biosensor regeneration without loss of protein ligand. Importantly, these experiments were performed on a biosensor that was already exposed to the acid regeneration buffer, so no increase in Antibody #1 binding signal is expected. (**A**) Sensorgram of ten Antibody #1 serial injections (blue traces) were performed with 60 s of 10 mM Glycine-HCl (pH = 2.4) acid regeneration (red bars) between each antibody injection. Complete return of the response (RU) signal back to baseline after acid regeneration indicates that all bound antibodies were successfully stripped prior to subsequent replicate injections. (**B**) Sensorgrams of Antibody #1 binding signal before (blue) and after (red) 35 cycles of acid regeneration. (**C**) Scatter plot comparing Antibody #1 binding signals measured from panel B between initial (blue) and final (red) antibody injections, following 35 acid regeneration cycles. The Y-axis plots R_max_ values of the first Antibody #1 injection, and the X-axis plots R_max_ values of the final Antibody #1 injection.

The HaloTag system leveraged for the capture of nascent IVTT proteins in the SPOC platform leads to covalent coupling between the expressed HaloTag enzyme and the chloroalkane ligand used to functionalize our protein capture substrates [28]. As such, we observe minimal ligand loss from SPOC biosensors while using harsh regeneration buffer formulations or when performing numerous regeneration injections over the course of long screens (Figure 5).

After the SPOC biosensor has been equilibrated with the 10 mM Glycine-HCl (pH = 2.4) acid regeneration buffer, we observe that Antibody #1 binding signals can be consistently reproduced, as in the case of 10 serial replicate injections of Antibody #1 (Figure 5A). The acid regeneration buffer completely strips away any bound Antibody #1 from the surface as indicated by the complete reset to baseline of the response (RU) signal after the sixty-secondregeneration buffer injections. We also tested the consistency of Antibody #1 binding response over long-term screening as in the case of a 24 h long experiment consisting of injection cycles with a total of 35 regeneration buffer injections, with Antibody #1 being injected first and last at the very end of the screening (Figure 5B). We observed excellent correlation (R-squared, R^2^ = 0.999) between Antibody #1 binding signals before and after up to 35 cycles of 10 mM Glycine-HCl (pH = 2.4) regeneration buffer injections (Figure 5C).

## 4. Discussion

Biomolecule interactions are not binary and are defined by a dynamic equilibrium between association and dissociation, which can have important implications for endpoint assays. Here, we illustrate how an interaction with fast kinetics can lead to false-negative results in fluorescent endpoint assay (Figure 2B). Furthermore, we highlight the advantages of real-time screening approaches like SPR, which can monitor transient interaction formation as they occur, reducing the risks of false-negative results (Figure 2D) and resolving the kinetic rates underlying biomolecule interactions (Figure 3), providing essential insights otherwise not readily attainable. In addition to kinetics, we highlight how it is vital to consider the conformational state of protein ligands when designing biomolecule interaction screening assays and when analyzing results (Figure 4).

**Figure 6:**
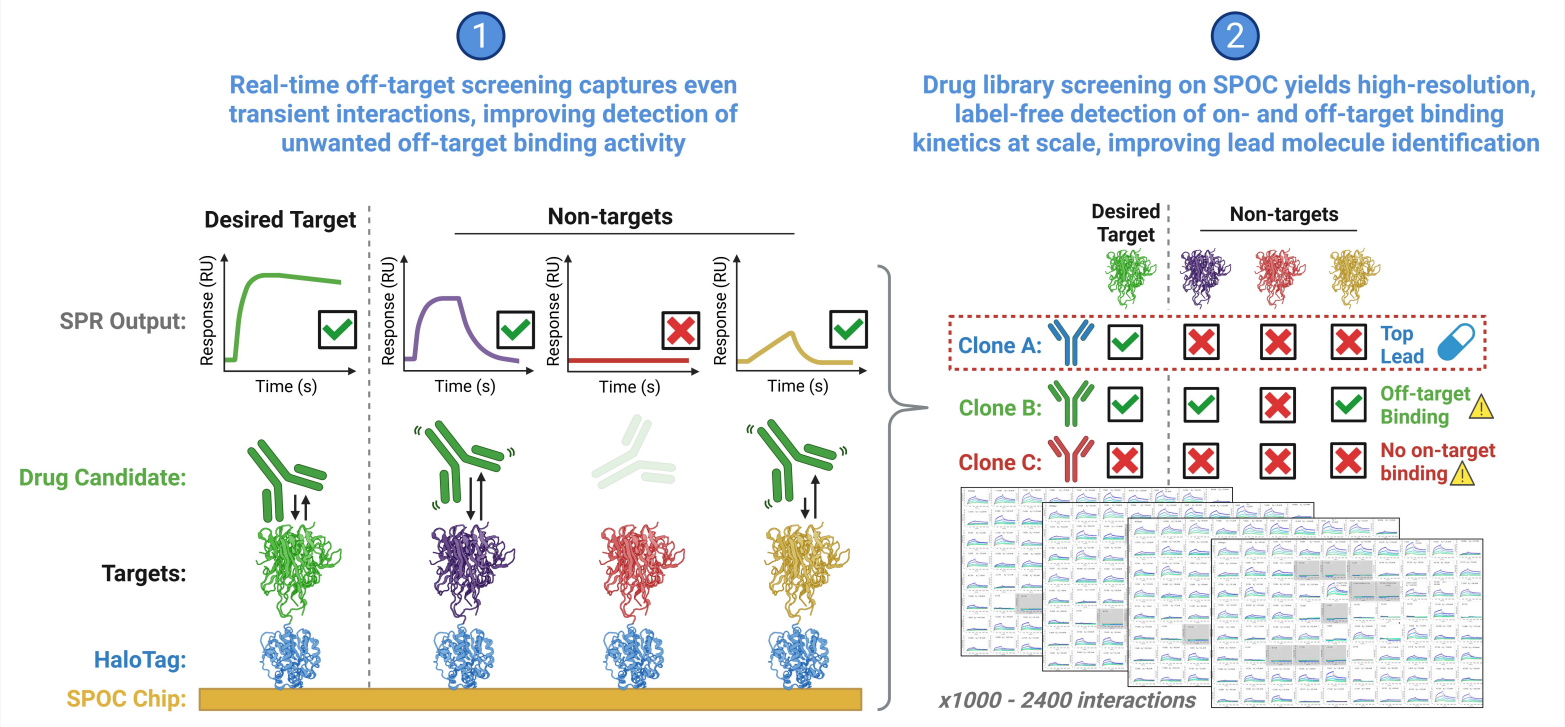
Graphical depiction SPOC technology applied to off-target screening in early drug discovery. A series of catalog chips is envisioned that display various proteins organized by target class, biochemistry, or specific applications as defined by the user, with up to 1000 to 2400 targets possible per chip. Desired chips would be synthesized cell-free and on-demand for off-target screening of therapeutic libraries or lead candidates by real-time SPR. On-target binding would be confirmed, and transient off-target interaction can be detected and flagged. Ultimately, lead molecules that perform the best can be identified and those with adverse promiscuity excluded.

For therapeutic development (Figure 6), SPOC real-time biosensing-based off-target interaction screening could advance secondary pharmacological screening regimens by improving the detection of potentially weaker but clinically significant off-target binding events otherwise missed in current endpoint screens utilized in the drug discovery process [34]. Binding of TPD drugs like PROTACs and molecular glues to neo-substrates is a well-recognized phenomenon and an issue underlying adverse drug reactions for this therapeutic modality [35,36]. Therefore, interrogating off-target binding events will be critical to assess as TPD-based therapies continue to be developed.

Beyond the advantages of real-time detection, the capability to assess the kinetic binding affinities of ternary complexes via SPOC would be of further value to the TPD field in identifying likely active compounds as correlations have been identified between ternary complex affinity and target degradation activity [34,37]. In the ADC space, ADRs are more complex and not just due to non-specific, off-target binding by the antibody, but they can also be due to factors such as on-target off-site binding to healthy tissue, payload linker stability and chemistry, non-specific ADC endocytosis due to overall antibody charge, and uptake into immune cells via Fcγ receptor binding [38]. In addition to off-target screening, SPOC platform could be leveraged to minimize the risk of ADRs by facilitating the screening of ADC designs that display moderate binding affinity to target antigens–a strategy being pursued to reduce off-site binding to healthy tissues that also express the target antigen, thereby reducing toxicity while maintaining efficacy [22]. An additional strategy to reduce off-site toxicity includes the design of target mutant-specific ADCs, and an SPOC assay harboring various mutant and wild-type target antigens could be implemented to screen libraries for variant-specific ADC candidates [39]. Finally, ADCs require endocytic uptake in order to impart lethal delivery of ADC payloads to cancerous tissue, a feature that can be drastically influenced by the precise epitope of the target against which any one particular ADC binds [40]. A future SPOC assay for ADC development pipelines could also include alanine-scanned variants of the target to facilitate down-selection of antibody libraries, yielding an antibody population with desired epitope-targeting diversity [41].

Leveraging SPR for diagnostic and point-of-care applications has been a long-term goal of the biosensing field, and portable devices have demonstrated equivalent performance to ELISA when performing serological screening for anti-SARS-CoV-2 antibodies in patient serum for example [42,43,44,45]. By converting traditional endpoint assays to real-time analyte detection, the risk of false-negative results could be reduced in an SPOC assay improving biomarker detection and diagnostic sensitivity. This advantage was recently highlighted in the case of an SPR assay for the detection and monitoring of anti-drug antibody (ADA) levels in patients that have received anti-TNFα therapeutic antibody treatment (Infliximab). It was observed that the real-time SPR-based screening outperformed endpoint ELISA when detecting these generally lower-affinity ADAs, which can nevertheless significantly reduce the efficacy of antibody therapies [46]. Furthermore, given that SPOC utilizes fully folded proteins and covalent capture chemistry, autoantibodies against both conformation and linear epitopes that have diagnostic utility—or antibodies used in other common orthogonal screening techniques where linear epitopes are assayed like immunohistochemistry (IHC)—can all be assessed [22].

## 5. Conclusions

Advances in label-free, real-time screening technologies have led to an expansion in the use of these approaches across industry and academia. The SPOC platform harnesses the improvements in cell-free expression and SPR technologies made over the past 30+ years and contributes the ability to generate high-content biosensors with hundreds of fully folded protein ligands in a customizable, affordable, and scalable fashion to address the screening and characterization needs of modern proteomics and drug discovery pipelines [26,47]. By increasing the base throughput of existing technologies manyfold, enabling the monitoring of up to 864 unique ligand interactions (with demonstrations of 2400 ligand capabilities), and leveraging in vitro transcription and translation from modular plasmid DNA to lower costs of full-length folded protein synthesis, we envision a range of applications for SPOC. For advanced diagnostics applications where a variety of interactions and ligand biochemistries may be employed (e.g., antibody fragments for biomarker detection alongside antigens for autoantibody detection), we envision grouping ligands into families with similar biochemistry and regeneration buffer requirements in distinct areas of the biosensor and employing flow cells with multiple channels to address these ligand families discretely so that optimal conditions can be applied, respectively. Furthermore, incorporating multichannel flow cells could be used to enhance the sample multiplexing such that multiple patient samples may be screened in parallel. In conclusion, real-time biosensing can overcome challenges when detecting interactions with fast kinetics, which can lead to false-negative results in traditional endpoint assays and limit their utility when detecting biomarkers in clinical screens or off-target binding hits in secondary pharmacological assays during drug development. SPOC is an emerging platform that seeks to make advances in these areas, leveraging innovations in cell-free protein expression and biosensing instrumentation enabling real-time interaction screening in an affordable, customizable, and highly multiplexed fashion.

## Data Availability

The authors confirm that all data supporting the findings of this study are provided within the article. Source data for the graphs and other inquiries can be requested and directed to the corresponding authors.

## Abbreviations

The following abbreviations are used in this manuscript:

SPOC	Sensor-integrated proteome on chip
SPR	Surface plasmon resonance
IVTT	In vitro transcription and translation
*k*_a_	Association rate
*k*_d_	Dissociation rate
*K*_D_	Equilibrium dissociation constant or binding affinity
*t*_1/2_	Half-life of bound complex
R_max_	Maximum binding response
CAR-T	Chimeric antigen receptor T-cell therapy
ADCs	Antibody drug conjugates
ADR	Adverse drug reaction
TPD	Targeted protein degradation
FDA	Food and Drug Administration
PBS	Phosphate-buffered saline
PBST-M	PBS containing Tween-20 and milk
RU	Response unit
ROI	Region of interest
BSA	Bovine serum albumin
